# Physical activity modification following a Transient Ischemic Attack in individuals with diabetes

**DOI:** 10.1186/s12933-024-02382-0

**Published:** 2024-08-07

**Authors:** Anastasios Mavridis, Tamar Abzhandadze, Adam Viktorisson, Katharina S. Sunnerhagen

**Affiliations:** 1 Institute of Neuroscience and Physiology, Rehabilitation Medicine, University of Gothenburg, Gothenburg, Sweden; 2 Department of Occupational Therapy and Physiotherapy, Sahlgrenska University Hospital, Gothenburg, Sweden; 3 Department of Rehabilitation Medicine, Neurocare, Sahlgrenska University Hospital, Vita stråket 12, fl. 4, 18 Sahlgrenska, 41345 Gothenburg Gothenburg, Sweden

**Keywords:** TIA, Exercise, Mortality, Cardiovascular, Lifestyle modification, Secondary prevention, Survival

## Abstract

**Background:**

Individuals with diabetes exhibit a higher risk of cardiovascular disease and mortality compared to healthy individuals. Following a transient ischemic attack (TIA) the risk of stroke and death increase further. Physical activity engagement after a TIA is an effective way of secondary prevention. However, there's a lack of research on how individuals with diabetes modify physical activity levels and how these adjustments impact survival post-TIA. This study aimed to determine the extent to which individuals with diabetes alter their physical activity levels following a TIA and to assess the impact of these changes on mortality.

**Methods:**

This was a nationwide longitudinal study, employing data from national registers in Sweden spanning from 01/01/2003 to 31/12/2019. Data were collected 2 years retro- and prospectively of TIA occurrence, in individuals with diabetes. Individuals were grouped based on decreasing, remaining, or increasing physical activity levels after the TIA. Cox proportional hazards models were fitted to evaluate the adjusted relationship between change in physical activity and all-cause, cardiovascular, and non-cardiovascular mortality.

**Results:**

The final study sample consisted of 4.219 individuals (mean age 72.9 years, 59.4% males). Among them, 35.8% decreased, 37.5% kept steady, and 26.8% increased their physical activity after the TIA. A subsequent stroke occurred in 6.7%, 6.4%, and 6.1% of individuals, while death occurred in 6.3%, 7.3%, and 3.7% of individuals, respectively. In adjusted analyses, participants who increased their physical activity had a 45% lower risk for all-cause mortality and a 68% lower risk for cardiovascular mortality, compared to those who decreased their physical activity.

**Conclusions:**

Positive change in physical activity following a ΤΙΑ was associated with a reduced risk of mortality. Increased engagement in physical activity should be promoted after TIA, thereby actively supporting individuals with diabetes in achieving improved health outcomes.

**Supplementary Information:**

The online version contains supplementary material available at 10.1186/s12933-024-02382-0.

## Background

Diabetes mellitus is a chronic metabolic disorder, affecting a substantial proportion of the global population [1]. In 1990, diabetes accounted for 1.96% of the global disease burden, while in 2019 this percentage had risen to 4.44% [2]. The global age-standardized prevalence of diabetes is constantly rising; from 3.2% in 1990 to 6.1% in 2021, with a projection to reach 9.8% by 2050 [1]. While mortality and cardiovascular disease (CVD) in individuals with diabetes have been declining [3], there still remains a higher risk for CVD and death compared to individuals without diabetes [4].

Transient Ischemic Attack (TIA) is defined by the American Heart Association as “a transient episode of neurological dysfunction caused by focal brain, spinal cord, or retinal ischemia, without acute infarction” [5]. While by definition, TIA leaves no lasting neurological deficits, it significantly increases the risk for a following cerebral infarction [6], as well as death [7]. Diabetes has been shown to increase the risk of TIA by 50% [8], and the risk of recurrent TIA or a subsequent ischemic stroke. It is also included in various risk stratification tools [9]. In order to minimize the risk of stroke and vascular death, an intensive multidisciplinary medical approach is required [10]. In addition, lifestyle modifications and, especially, physical activity engagement should be implemented in the care of TIA patients [10]. Consequently, it should be the case that, following a TIA, patients increase their physical activity levels.

In patients with diabetes, low physical activity has been associated with increased all-cause mortality [11, 12]. Furthermore, it has been shown that exercise interventions reduce CVD risk factors following a stroke or TIA [13], while another study showed that stroke survivors do not meet physical activity guidelines post-stroke [14]. However, considering the non-disabling nature of TIAs, physical activity after TIA should be investigated separately. We have identified one qualitative study addressing how individuals modify their physical activity following a TIA [15], where it was found that no significant changes are made.

To the best of our knowledge, there are no studies that explore if individuals with diabetes modify their physical activity after a TIA. This study aims to identify how individuals with diabetes change their physical activity levels following a TIA and explore if these modifications are associated with post-TIA all-cause and CVD-specific mortality. The objective of this study is to evaluate the effectiveness of physical activity interventions for secondary prevention following a TIA, providing valuable insights for optimizing patient care and outcomes in this high-risk population.

## Methods

This study followed the guidelines for Strengthening the Reporting of Observational Studies in Epidemiology (STROBE) [16].

### Study design and data sources

This was a nationwide longitudinal study, employing data from national registers in Sweden. The National Diabetes Register (NDR) was used for the collection of the study population. The NDR includes the vast majority of individuals with diabetes in Sweden with a reported coverage of 88% in 2019 [17]. The register includes clinical and laboratory data from regular follow-up visits of adults with diabetes and detailed information can be found elsewhere [18]. The Swedish National Patient Register (NPR) [19] was used for the selection of individuals that were diagnosed with a TIA (the International Classification of Diseases, Tenth Revision code [ICD-10], G45.9), as well as for collection of data on comorbid conditions. This register includes diagnoses received by the patients during inpatient and specialized outpatient care. Data on education and income were collected from the Statistics Sweden database [20]. Mortality rates, causes, and dates of death were collected from the Swedish Cause of Death register [21]. The National Board of Health and Welfare in Sweden was responsible for data linkage and pseudonymization of files that were sent to the researchers.

The study population consisted of individuals diagnosed with TIA after their first entry into the NDR and between 01/01/2005 and 31/12/2017. Individuals were tracked both retrospectively and prospectively for up to two years from the occurrence of TIA. Therefore, the study period included TIA diagnoses between 01/01/2005 and 31/12/2017, while NDR registrations ranged between 01/01/2003 and 31/12/2019. Individuals with a stroke diagnosis prior to TIA or a TIA diagnosis after their last NDR registration were excluded from the study.

### TIA diagnosis

According to the National Register of stroke care quality of Sweden (Riksstroke) [22], in 2017, 97% of TIA patients were examined with computed tomography (CT), and 12% were examined with magnetic resonance imaging (MRI). A total 98% of TIA patients were examined with either CT or MRI [22]. The definition of TIA was time-based; patients with complete symptom regression within 24 h should be registered as TIA patients [22].

### Physical activity assessment

Physical activity was self-reported by the individual on each follow-up. It was assessed on a 5-point scale based on how many times per week the individual engaged in at least 30 min of physical activity with an intensity equivalent to outdoor walking. The scale consists of the following levels:

Level 1: Never.

Level 2: < 1 times per week.

Level 3: 1–2 times per week.

Level 4: 3–5 times per week.

Level 5: 6–7 times per week.

### Outcome

The main outcome of this study was all-cause mortality, CVD mortality, and non-CVD mortality. CVD mortality was defined as death due to disease of the circulatory system, as included in the ICD-10 codes I00-I99. Non-CVD mortality was defined as death due to any other cause (all ICD-10 codes except I00-I99). Mortality rates were followed from the occurrence of TIA until death or censoring 2 years after the TIA.

### Covariates

The following covariates were collected and analysed.

Sociodemographic variables: Age on diagnosis of TIA, sex, diabetes type, smoking status 2 years prior and after TIA, education level the year before the TIA (classified as lower [≤ 9 years], secondary [10– 11 years], and higher education [≥ 12 years]), and disposable family income the year before the TIA.

Pre-existing comorbidities: Atrial fibrillation, myocardial infarction, heart failure, and chronic respiratory conditions were considered present if they were diagnosed at any time before TIA occurrence. Cancer, substance abuse and peripheral vascular disease were considered present if they were diagnosed no more than 2 years prior to TIA.

Clinical and laboratory data: Glycated haemoglobin (HbA1c), systolic and diastolic blood pressure, body mass index (BMI), total cholesterol, triglycerides, high density lipoprotein (HDL), low density lipoprotein (LDL), and GFR. These covariates were included separately before and after the TIA, and they are measured as mean values from all registrations for a maximum of 2 years pre- and post-TIA.

Subsequent CVD events: Occurrence of stroke (both ischemic and haemorrhagic) and recurrent TIA, if they occurred within 2 years from the first TIA.

## Statistical analyses

### Physical activity change

The median level of physical activity was calculated from all NDR registrations for a maximum of 2 years before the first TIA diagnosis and for 2 years after, or until death, whichever occurred first. The individuals were then categorized into three groups: decreasing, steady, or increasing physical activity, based on if the difference between post-TIA and pre-TIA physical activity was negative, zero, or positive, respectively.

### Survival analyses

Cox proportional hazards models were employed to assess the adjusted relationship between mortality and physical activity modification following a TIA. Separate models were created for all-cause mortality, CVD mortality and non-CVD mortality. In each model the included covariates were sex, age on TIA, smoking status prior to TIA, number of comorbidities, stroke occurrence and recurrent TIA during the 2-year follow-up, and physical activity group (increase, steady, decrease). The models were also adjusted for the level of physical activity prior to TIA, since physical activity modification cannot be evaluated independently of the prior physical activity level. The proportional hazards assumption was checked using scaled Schoenfeld residuals, and it was fulfilled for all variables, as well as globally for all models.

### Sensitivity analysis

In the primary analysis based on complete cases, three key variables (pre-TIA physical activity, post-TIA physical activity, and smoking status pre-TIA) exhibited a combined 37.6% missing data rate. This substantial level of missing data could potentially impact the results. Observing the patterns of the missing data suggested that it was not missing completely at random. However, we could not definitively classify the missing data mechanism as solely'missing at random' or'missing not at random'.

To increase the validity of our results, multiple imputation using predictive mean matching was performed [23]. The variables pre-TIA physical activity, post-TIA physical activity, and smoking status pre-TIA were imputed and used as predictors. All other variables in the dataset were used only as predictors (sex, age on TIA, diabetes type, pre-TIA clinical and laboratory values [except physical activity and smoking], post-TIA clinical and laboratory values [except physical activity], stroke occurrence, TIA recurrence and death with their corresponding time to event, presence of each comorbidity, cause of death, education, and income). Ten imputations were performed. Following that, adjustments were made to calculate changes in physical activity, assign groups based on physical activity change, and conduct survival analyses for all-cause, CVD and non-CVD mortality as described in the main analysis (online-only supplement).

All statistical tests were two tailed performed at alpha 5%. Statistical analyses were carried out using R (R Core Team [2023], R: A Language and Environment for Statistical Computing, R Foundation for Statistical Computing, Vienna, Austria.) and SPSS (IBM SPSS Statistics for Windows, Version 29.0, released 2022; IBM Corp., Armonk, NY).

## Results

### Study sample

**Fig. 1:**
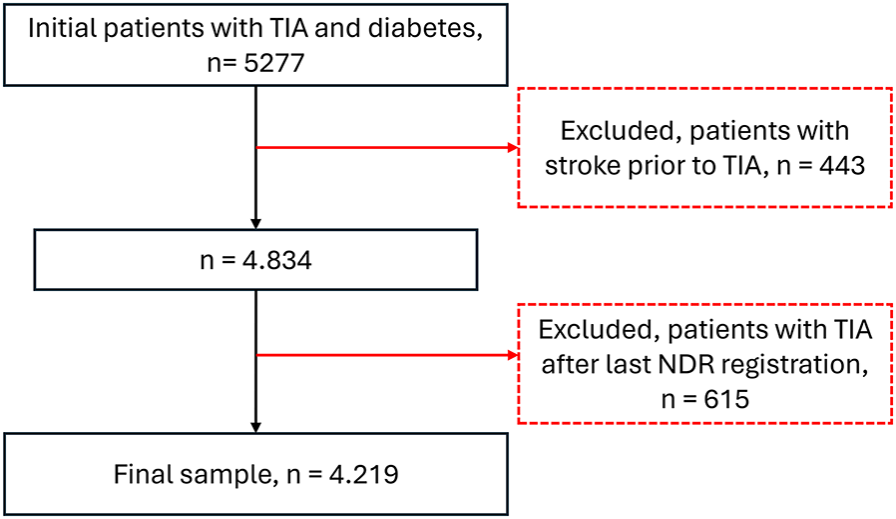
Flowchart of the study sample

The initial number of individuals that received a TIA diagnosis after their first NDR registration and between 01/01/2005 and 31/12/2017 was 5277. After excluding individuals with a diagnosis of stroke prior to TIA (n = 443) and individuals with a TIA diagnosis after their last NDR registration (n = 615), the final study sample consisted of 4219 individuals (Fig. 1). The median number of physical activity registrations for each individual was 6 (3 before and 3 after the TIA).

### Physical activity modification

The individuals were categorized in three groups based on their physical activity levels before and after the TIA (Tables 1, 2). The decrease group (n = 971, 35.8%) showed a reduction from a median physical activity level of 4 to 2. The steady group (n = 1017, 37.5%) remained at a median physical activity level of 4 before and after TIA. The increase group (n = 726, 26.8%) showed an increase from a median physical activity of 3 to 4. The changes of physical activity level for the total sample are presented in Supplementary Fig. 1.A.

**Table 1:** Descriptive characteristics stratified by group of physical activity change and for total sample

	Physical activity change^1^			
	Decrease, n (valid %)	Stable, n (valid %)	Increase, n (valid %)	Total, n (valid %)
	971 (35.8)	1017 (37.5)	726 (26.8)	4219 (100.0)
Age at TIA^2^ mean, (SD)	73.6 (9.9)	72.7 (10.3)	71.2 (10.5)	72.9 (10.6)
**Sex**^**3**^				
Male	585 (60.2)	628 (61.8)	444 (61.2)	2506 (59.4)
Female	386 (39.8)	389 (38.2)	282 (38.8)	1713 (40.6)
**Diabetes type**^**4**^				
Type 1	66 (6.8)	81 (8.0)	51 (7.0)	271 (6.4)
Type 2	899 (92.6)	928 (91.2)	672 (92.6)	3899 (92.6)
Other//unknown type	6 (0.6)	8 (0.8)	3 (0.4)	39 (0.9)
**Comorbidities pre-TIA, yes**^**5**^				
Cancer	105 (10.8)	94 (9.2)	65 (9.0)	407 (9.6)
Alcohol or substance abuse	8 (0.8)	10 (1.0)	5 (0.7)	38 (0.9)
Hypertension	453 (46.7)	473 (46.5)	343 (47.2)	1933 (45.8)
Atrial fibrillation	131 (13.5)	125 (12.3)	81 (11.2)	547 (13.0)
Myocardial infarction	18 (1.9)	30 (2.9)	23 (3.2)	102 (2.4)
Heart failure	77 (7.9)	62 (6.1)	31 (4.3)	285 (6.8)
Respiratory conditions	79 (8.1)	54 (5.3)	48 (6.6)	269 (6.4)
Rheumatic conditions	78 (8.0)	79 (7.8)	63 (8.7)	322 (7.6)
Peripheral vascular disease	58 (6.0)	58 (5.7)	36 (5.0)	240 (5.7)
**Outcomes, yes**				
Stroke^6^	65 (6.7)	65 (6.4)	44 (6.1)	251 (5.9)
Recurrent TIA^7^	147 (15.1)	170 (16.7)	115 (15.8)	660 (15.6)
Death^8^	61 (6.3)	74 (7.3)	27 (3.7)	343 (8.1)
**Education**^**9**^				
Lower (≤ 9 years)	418 (43.5)	426 (42.3)	276 (38.6)	1753 (42.2)
Secondary (10– 11 years)	276 (28.8)	295 (29.3)	209 (29.2)	1211 (29.1)
Higher (≥ 12 years)	266 (27.7)	286 (28.4)	230 (32.2)	1191 (28.7)
**Income**^**10**^**,** thousands of Swedish crowns				
Lower ≤ 1886	301 (31.0)	304 (29.9)	232 (32.0)	1408 (33.4)
Middle 1887– 3120	340 (35.0)	360 (35.4)	219 (30.2)	1405 (33.3)
High > 3120	330 (34.0)	353 (34.7)	275 (37.9)	1406 (33.3)

Missing values 1: 1505, 2: 0, 3: 0, 4: 10, 5: 0, 6: 0, 7: 0, 8: 0, 9: 64, 10: 0

TIA: Transient Ischemic Attack, SD: Standard Deviation

**Table 2:** Clinical and Laboratory values, stratified by group of physical activity change and for total sample

	Physical activity change							
	Decrease, Mean (SD)		Stable, Mean (SD)		Increase, Mean (SD)		Total, Mean (SD)	
	Pre-TIA	Post-TIA	Pre-TIA	Post-TIA	Pre-TIA	Post-TIA	Pre-TIA	Post-TIA
Physical Activity^1^, Median (IQR)	4.0 (2.0)	2.0 (2.0)	4.0 (3.0)	4.0 (3.0)	3.0 (1.5)	4.0 (2.0)	3.5 (2.5)	3.0 (2.5)
HbA1c^2^, mmol/L	55.6 (12.6)	56.2 (12.5)	55.3 (12.2)	55.4 (11.9)	56.2 (12.3)	55.3 (11.8)	55.7 (12.8)	56.0 (12.9)
Systolic BP^3^, mmHg	136.6 (14.0)	134.1 (12.6)	137.0 (14.0)	134.6 (13.5)	136.9 (14.2)	134.5 (13.1)	137.4 (14.6)	135.1 (14.0)
Diastolic BP^4^, mmHg	74.6 (8.0)	73.4 (8.2)	75.6 (8.4)	73.9 (8.2)	75.6 (8.5)	74.2 (7.8)	75.4 (8.5)	74.1 (8.4)
BMI^5^, kg/m2	28.8 (4.6)	28.4 (4.8)	28.9 (5.0)	28.5 (4.9)	29.1 (4.9)	28.7 (4.9)	28.9 (4.9)	28.5 (4.9)
Triglycerides^6^, mmol/L	1.8 (1.0)	1.7 (0.9)	1.8 (1.2)	1.7 (1.0)	1.8 (1.1)	1.7 (1.1)	1.8 (1.1)	1.7 (1.0)
LDL^7^, mmol/L	2.7 (1.0)	2.3 (0.9)	2.6 (0.9)	2.3 (0.8)	2.6 (1.0)	2.3 (0.9)	2.7 (0.9)	2.4 (0.9)
GFR^8^, mL/min/1.73m2	73.2 (22.2)	71.2 (23.7)	74.7 (21.9)	72.6 (23.1)	75.8 (21.4)	73.7 (22.2)	74.0 (22.2)	72.0 (23.8)
Total Cholesterol^9^, mmol/L	4.7 (1.0)	4.3 (1.0)	4.6 (1.0)	4.3 (1.0)	4.7 (1.2)	4.2 (1.0)	4.7 (1.1)	4.3 (1.0)
HDL^10^, mmol/L	1.3 (0.4)	1.3 (0.4)	1.3 (0.4)	1.3 (0.4)	1.3 (0.4)	1.3 (0.4)	1.3 (0.4)	1.3 (0.4)
Smoking^11^, yes, n (valid %)	126 (13.5)	114 (12.3)	110 (11.1)	103 (10.4)	106 (15.0)	90 (12.7)	504 (14.0)	436 (12.1)

Missing values pre-TIA: 1: 990, 2: 340, 3: 379, 4: 383, 5: 670, 6: 1347, 7: 1181, 8: 548, 9: 924, 10: 1194,11: 609

Missing values post-TIA: 1: 948, 2: 318, 3: 349, 4: 349, 5: 657, 6: 1382, 7: 1091, 8: 499, 9: 914, 10: 1140,11: 614

SD: Standard Deviation, TIA: Transient Ischemic Attack IQR: Interquartile range, HbA1c: Glycated haemoglobin, BP: Blood Pressure, BMI: Body Mass Index, LDL: Low Density Lipoprotein, GFR: Glomerular Filtration Rate, HDL: High Density Lipoprotein

### Descriptive characteristics

Descriptive characteristics, stratified by physical activity groups and for the total study sample are presented in Table 1. The group which increased their physical activity post-TIA exhibited the lowest stroke incidence (6.1%) and lowest mortality (3.7%). The change of clinical and laboratory variables pre- and post-TIA stratified by groups of physical activity change and for total sample are presented in Table 2.

### Survival analyses

**Fig. 2:**
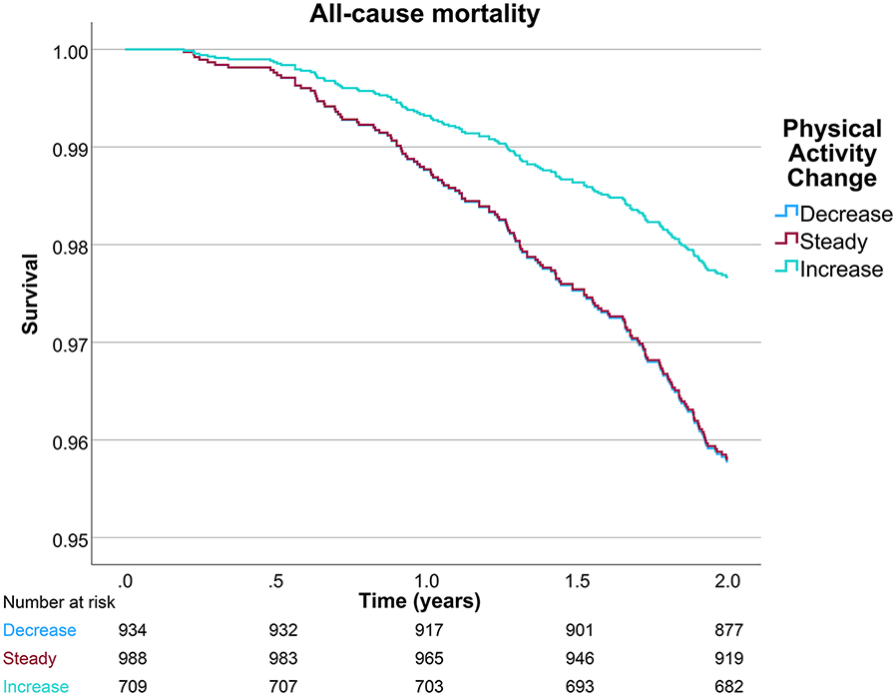
Survival plot of cox regression analysis of all-cause mortality following a TIA

**Fig. 3:**
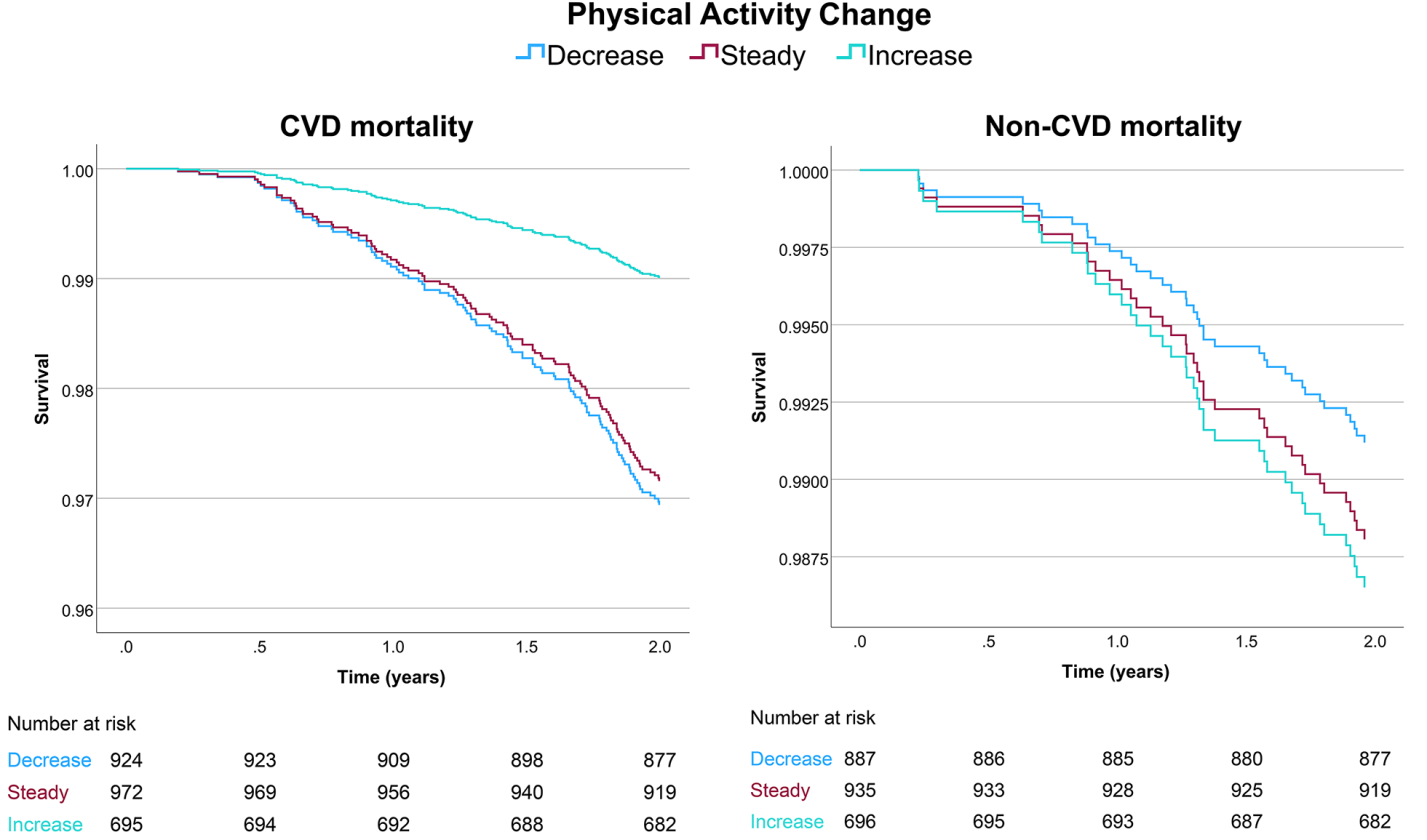
Survival plot of cox regression analyses of CVD and non-CVD mortality following a TIA

During 2 years of follow-up the groups that decreased, kept stable, and increased their physical activity following a TIA exhibited 6.3%, 7.3%, and 3.7% crude all-cause mortality, respectively. The results of the Cox proportional hazards models for all-cause, CVD, and non-CVD mortality are presented in Table 3. Individuals who increased their physical activity after a TIA exhibited 45% (HR 0.55, 95% CI 0.34–0.89) lower 2-year risk for all-cause mortality, compared to individuals who decreased their physical activity. For CVD specific 2-year mortality, risk-reduction reached 68% (HR 0.32, 95% CI 0.17–0.61) for the same group. For non-CVD mortality, physical activity change was not significantly associated with mortality. The survival plots of the 3 models stratified by groups of physical activity are presented in Fig. 2 for all-cause mortality and Fig. 3 for CVD and non-CVD mortality.

**Table 3:** Cox proportional hazards models for all-cause, CVD and non-CVD mortality following a Transient Ischemic Attack

	All-cause mortality		CVD mortality		Non-CVD mortality	
	HR (95% CI)	p-value	HR (95% CI)	p-value	HR (95% CI)	p-value
Group of physical activity change		.022		.001		.620
Decrease (Reference)						
Stable	0.99 (0.69–1.44)	.976	0.93 (0.61–1.41)	.727	1.36 (0.60–3.09)	.466
Increase	0.55 (0.34–0.89)	.015	0.32 (0.17–0.61)	<.001	1.54 (0.64–3.67)	.334
Female sex	0.61 (0.43–0.86)	.005	0.63 (0.42–0.94)	.023	0.51 (0.26–1.03)	.060
Age at TIA (range 22–105)	1.10 (1.07–1.12)	<.001	1.11 (1.08–1.14)	<.001	1.08 (1.03–1.12)	<.001
Physical activity pre-TIA	0.79 (0.70–0.90)	<.001	0.78 (0.68–0.90)	<.001	0.79 (0.62–1.01)	.057
Smoking pre-TIA	1.72 (1.04–2.85)	.034	2.00 (1.13–3.55)	.018	1.18 (0.40–3.44)	.765
Stroke	1.84 (1.09–3.08)	.022	2.38 (1.38–4.10)	.002	0.44 (0.06–3.18)	.412
Recurrent TIA	0.77 (0.49–1.22)	.265	0.66 (0.38–1.16)	.153	1.08 (0.48–2.46)	.847
Number of comorbidities (range 0–7)	1.27 (1.11–1.45)	<.001	1.36 (1.18–1.58)	<.001	1.02 (0.76–1.36)	.920

All-cause mortality: Events: 153, Total: 2631, -2 Log Likelihood: 2262, χ^2^: 125.4, p < 0,001

CVD mortality: Events: 113, Total: 2591, -2 Log Likelihood: 1634, χ^2^: 124.4, p < 0,001

Non-CVD mortality: Events: 40, Total: 2518, -2 Log Likelihood: 599, χ^2^: 24.2, p = 0,004

CVD: Cardiovascular disease, HR: Hazard Ratio, CI: Confidence Interval, TIA: Transient Ischemic Attack

### Sensitivity analysis

The distributions of pre- and post-TIA physical activity in the original and the imputed datasets are presented in Supplementary Fig. 2. The changes of physical activity level for the total sample after imputation are presented in Supplementary Fig. 1.B. Pooled estimates of cox proportional hazards models for all-cause, CVD and non-CVD mortality from 10 imputed datasets are presented in Supplementary Table 1. Physical activity increase remained a significant factor for CVD mortality, with a risk reduction of 56% (HR 0.44, 95% CI 0.21–0.93) compared to physical activity decrease, but significance did not hold for all-cause or non-CVD mortality.

## Discussion

This longitudinal nationwide study aimed to identify how individuals with diabetes modify their physical activity level following a TIA, and how this modification was associated with 2-year mortality risk. We found that more than 37% of TIA patients with diabetes did not modify their physical activity, while more than 35% of them decreased it. The participants that increased their physical activity level exhibited a lower frequency of subsequent stroke and death in the 2 years following a TIA. These results indicate that even though an increase in physical activity after a TIA can substantially improve outcomes, the majority of participants did not increase their physical activity engagement.

Following a TIA, lifestyle modifications and physical activity engagement are necessary measures for secondary prevention [10]. A qualitative study of individuals with TIA and mild stroke, found that physical activity following a TIA is not adequately increased, specifically it was characterized “business as usual” [15]. Our study, even though focused on individuals with diabetes, affirms these results, with 73% of the participants not increasing or even decreasing their physical activity level. This finding raises concerns regarding the efficiency of patient motivation and adequate education on the importance of physical activity in secondary prevention following a TIA.

Several important differences were observed between the three groups of physical activity change in their baseline characteristics. Those who increased their physical activity were younger and had a lower prevalence of heart failure and respiratory conditions compared to those who decreased their activity. Although these differences could influence physical activity levels, it is notable that the group with decreased activity had a higher level of pre-TIA physical activity. Furthermore, there were no clinically significant differences in laboratory values before and after TIA among the three groups. This finding is unexpected, as we typically anticipate improvements in these markers due to the medications prescribed following a TIA. However, we believe this lack of difference may be because individuals with diabetes are regularly monitored. Consequently, markers such as blood pressure, LDL, HDL, and HbA1c are consistently maintained within normal ranges through appropriate medication adjustments.

In this study, a lower frequency of subsequent stroke and mortality after a TIA, was observed in the group which increased their physical activity. High level of physical activity has been associated with decreased risk of CVD and mortality, both in individuals with diabetes [3, 11, 12, 24–27] and in the general population [28]. Interestingly, the group of participants that decreased their physical activity, exhibited substantially higher mortality, even though they initiated from a higher baseline. In adjusted analyses, physical activity modification was independently associated with mortality. Specifically, the strongest association was observed in CVD mortality, with these results being confirmed in sensitivity analysis, using imputed data. Notably, the benefit of physical activity increase on survival was independent from the baseline level of physical activity. These findings indicate that even if the pre-TIA physical activity level is low, increasing physical activity post-TIA may substantially reduce all-cause and CVD mortality risk. A similar association between physical activity increase, independent of baseline, and mortality, was found by a large population study, although there was no focus on TIA [29]. Furthermore, it is notable that the steady group maintained a high level of physical activity both before and after TIA, whereas the increasing group reached this level only after the event. Despite this, the mortality remained higher in the steady group. Various explanations can be given to these results. Firstly, age is undeniably an important factor with the steady group being, on average, 1.5 years older. Additionally, it's plausible that the increase in physical activity in the increasing group is indicative of broader lifestyle improvements they undertook post-TIA, such as smoking cessation, adhering more strictly to medication regimens, and adopting a healthier diet, with physical activity being just one aspect of their overall commitment to well-being. Lastly, it might also be the case that improving physical activity independently impacts mortality, regardless of the specific level of physical activity.

Changing entrenched behaviours and promoting healthy lifestyles can be challenging, yet increasing physical activity remains crucial [10]. While advising patients on lifestyle improvements is important, it is often insufficient without accompanying behavioural interventions [30]. Combining exercise-based and behaviour change programs is more effective in reducing stroke risk factors [30]. Targeted secondary prevention programs, such as cardiac rehabilitation programs or exercise and lifestyle counselling, tailored to the disabilities, capacities, and preferences of each patient, have shown significant benefits [31, 32]. These comprehensive interventions not only address physical activity but also support broader lifestyle changes, ultimately enhancing patient outcomes.

This study has several strengths and limitations. The use of large register data allowed us to include the vast majority of individuals with diabetes that suffered a TIA in Sweden. Furthermore, with the use of the NDR we acquired longitudinal data on a variety of clinical and laboratory variables, thereby increasing the reliability of the data and the robustness of the analysis. Some limitations exist regarding the physical activity variable used in this study, which relied on self-reporting, potentially introducing reporting bias. However, objectively measuring physical activity on a population level poses significant challenges, making self-reporting an imperfect, yet necessary approach in this context. All types of diabetes were analysed, but the small number of non-type 2 participants was not sufficient for separate reliable results. However, despite differing pathophysiological development, both type 1 and type 2 diabetes share similar risk factors for CVD [33]. Lastly, it should be noted that a significant portion of the sample lacked data on pre-TIA physical activity, smoking status, and post-TIA physical activity, all of which were used in survival analyses. Handling missing register data in mortality analysis can be challenging, since the mechanism of the missingness cannot be assumed. Consequently, no single statistical approach could adequately handle the missing data [34]. Given these challenges, we conducted both complete-case analysis and multiple imputation. Both analyses showed that at least for CVD mortality physical activity modification remains a significant factor. In general, we assert that the results of the present study, hold generalizability in individuals with diabetes in western countries, with publicly accessible, tax-funded healthcare systems similar to Sweden’s. However, it is crucial to interpret these findings cautiously, recognizing that they indicate associations rather than establish causation.

## Conclusions

In conclusion, this study highlights the concerning trend of limited physical activity increase among individuals with diabetes following a TIA. Despite the benefits of lifestyle changes post-TIA, a substantial proportion of patients do not increase their physical activity levels, with some even decreasing engagement. However, those who did increase their activity levels experienced lower subsequent stroke and mortality rates. The protective impact of increasing physical activity becomes more evident in CVD related mortality. Even small changes in physical activity can be beneficial, regardless of pre-TIA physical activity level, highlighting the need for improvement in this aspect of TIA care. Given the "invisible" nature of TIAs, which typically leave no lasting neurological deficits or disabilities, it becomes crucial to thoroughly educate patients about the risks associated with TIAs and the critical role of secondary prevention through both medication and lifestyle adjustments. Individualized physical activity plans tailored to each patient's needs and capabilities are essential to enhance motivation, ensure adherence, and ultimately improve survival rates.

### Supplementary Information

Supplementary material 1

## Data Availability

According to the Swedish regulations http://www.epn.se/en/start/regulations/, data can only be used in accordance with the application for this study that was approved by the Ethical board. Requests to access the dataset can be submitted by qualified researchers to the authors (contact Professor Katharina S. Sunnerhagen, email: ks.sunnerhagen@neuro.gu.se).

## Abbreviations

BMI: Body Mass Index
CVD: Cardiovascular Disease
GFR: Glomerular Filtration Rate
HbA1c: Glycated Haemoglobin
HDL: High Density Lipoprotein
ICD-10: The International Classification of Diseases, Tenth Revision
LDL: Low Density Lipoprotein
NDR: National Diabetes Register
NPR: National Patient Register
TIA: Transient Ischemic Attack
