# Supplementary material for: Physical activity modification following a Transient Ischemic Attack in individuals with diabetes
Source: Cardiovasc Diabetol. 2024 Aug 7;23:288. doi: 10.1186/s12933-024-02382-0 (PMC11304772; doi:10.1186/s12933-024-02382-0)
Supplement: Supplementary file 1 — Supplementary material 1 [file 12933_2024_2382_MOESM1_ESM.docx]

**Title:**

Physical activity modification following a Transient Ischemic Attack in individuals with diabetes

**Authors:**

Anastasios Mavridis, Tamar Abzhandadze, Adam Viktorisson, Katharina S. Sunnerhagen

**Affiliations:**

Institute of Neuroscience and Physiology, Rehabilitation Medicine, University of Gothenburg, Sweden

Department of Rehabilitation medicine, Neurocare, Sahlgrenska University Hospital, Gothenburg, Sweden

Department of Occupational Therapy and Physiotherapy, Sahlgrenska University Hospital, Gothenburg, Sweden

**Corresponding author:** Tamar Abzhandadze, Rehabiliteringsmedicin, Vita stråket 12, fl. 4, Sahlgrenska universitetssjukhuset, 41345 Gothenburg. +467022622897, [tamar.abzhandadze@gu.se](mailto:tamar.abzhandadze@gu.se)

# Contents

**Supplementary Figure 1** – Sankey diagram depicting the changes of physical activity following a Transient Ischemic Attack for the original (A) and the imputed (B) data.

**Supplementary Figure 2** - Comparison of pre-TIA (A) and post-TIA (B) physical activity distribution in the original data and the imputed data.

**Supplementary Table 1** - Pooled estimates from 10 imputations of missing data. Cox proportional hazards models for all-cause, CVD and non-CVD mortality following a Transient Ischemic Attack in individuals with diabetes.


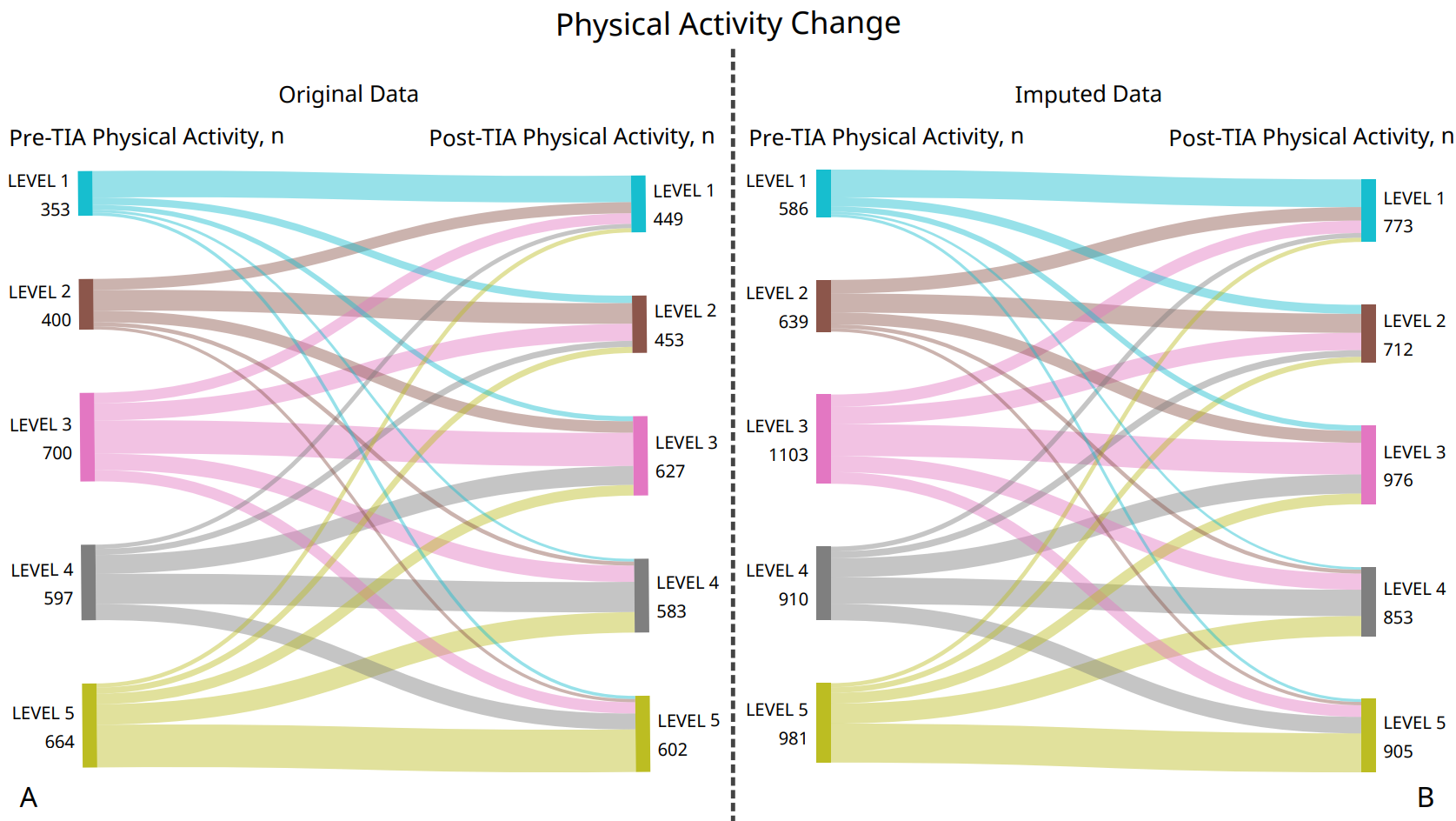


Supplementary Figure 1. Sankey diagram depicting the changes of physical activity following a Transient Ischemic Attack for the original (A) and the imputed (B) data. Level of physical activity shows how many times per week the individual engaged in at least 30 minutes of physical activity with an intensity equivalent to outdoor walking: Level 1: Never, Level 2: <1 times, Level 3: 1-2 times, Level 4: 3-5 times, Level 5: 6-7 times.


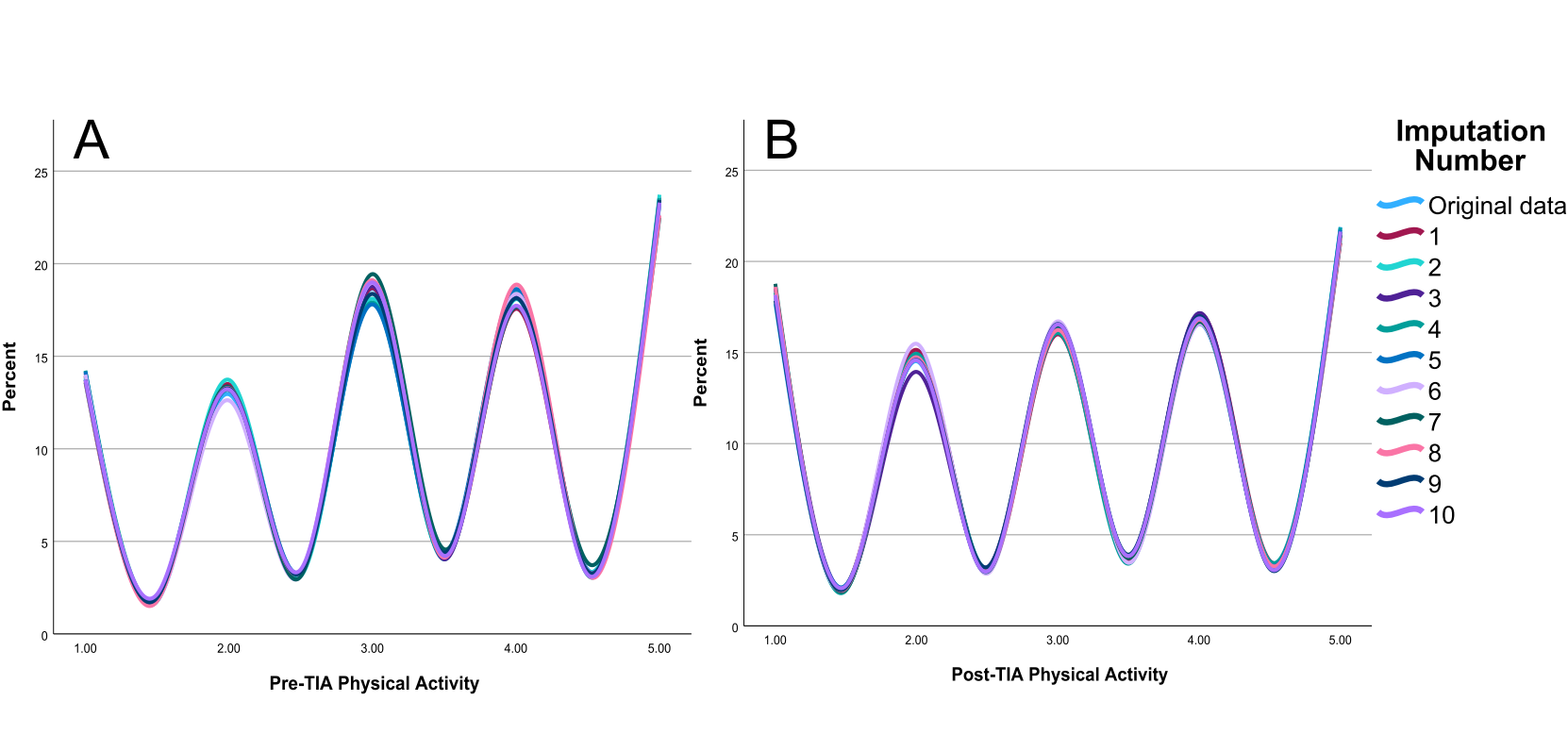


Supplementary Figure 2. Comparison of pre-TIA (A) and post-TIA (B) physical activity distribution in the original data and the imputed data.

Supplementary Table 1. Pooled estimates from 10 imputations of missing data. Cox proportional hazards models for all-cause, CVD and non-CVD mortality following a Transient Ischemic Attack in individuals with diabetes.

|  | All-cause mortality | | CVD mortality | | Non-CVD mortality | |
| --- | --- | --- | --- | --- | --- | --- |
|  | HR (95% CI) | p-value | HR (95% CI) | p-value | HR (95% CI) | p-value |
| Group of physical activity change |  |  |  |  |  |  |
| Decrease (Reference) |  |  |  |  |  |  |
| Stable | 0.87 (0.62-1.23) | 0.423 | 0.84 (0.56-1.25) | 0.369 | 1.02 (0.48-2.16) | 0.959 |
| Increase | 0.61 (0.36-1.04) | 0.066 | 0.44 (0.21-0.93) | 0.034 | 1.33 (0.64-2.77) | 0.444 |
| Female sex | 0.71 (0.57-0.89) | 0.003 | 0.73 (0.56-0.94) | 0.013 | 0.61 (0.38-0.98) | 0.040 |
| Age on TIA (range 22-105) | 1.10 (1.08-1.11) | <0.001 | 1.11 (1.09-1.12) | <0.001 | 1.08 (1.05-1.11) | <0.001 |
| Physical activity pre-TIA | 0.81 (0.74-0.90) | <0.001 | 0.79 (0.70-0.88) | <0.001 | 0.89 (0.73-1.09) | 0.248 |
| Smoking pre-TIA | 1.92 (1.37-2.69) | <0.001 | 2.24 (1.54-3.26) | <0.001 | 1.13 (0.51-2.52) | 0.767 |
| Stroke | 1.32 (0.89-1.95) | 0.171 | 1.60 (1.06-2.42) | 0.026 | 0.43 (0.10-1.73) | 0.232 |
| Recurrent TIA | 0.79 (0.58-1.09) | 0.152 | 0.76 (0.52-1.09) | 0.135 | 0.90 (0.49-1.66) | 0.732 |
| Number of comorbidities (range 0-7) | 1.27 (1.17-1.39) | <0.001 | 1.33 (1.20-1.46) | <0.001 | 1.12 (0.92-1.36) | 0.250 |
| All-cause mortality: Events: 343, Total: 4219  CVD mortality: Events: 261, Total: 4137  Non-CVD mortality: Events: 82, Total: 3958  CVD: Cardiovascular disease, HR: Hazard Ratio, CI: Confidence Interval, TIA: Transient Ischemic Attack | | | | | | |
